# Long Non-coding RNA in Neuronal Development and Neurological Disorders

**DOI:** 10.3389/fgene.2018.00744

**Published:** 2019-01-23

**Authors:** Ling Li, Yingliang Zhuang, Xingsen Zhao, Xuekun Li

**Affiliations:** ^1^The Children’s Hospital, School of Medicine, Zhejiang University, Hangzhou, China; ^2^Institute of Translational Medicine, School of Medicine, Zhejiang University, Hangzhou, China

**Keywords:** long non-coding RNA, gene expression, neuronal development, neurological disorders, mechanism

## Abstract

Long non-coding RNAs (lncRNAs) are transcripts which are usually more than 200 nt in length, and which do not have the protein-coding capacity. LncRNAs can be categorized based on their generation from distinct DNA elements, or derived from specific RNA processing pathways. During the past several decades, dramatic progress has been made in understanding the regulatory functions of lncRNAs in diverse biological processes, including RNA processing and editing, cell fate determination, dosage compensation, genomic imprinting and development etc. Dysregulation of lncRNAs is involved in multiple human diseases, especially neurological disorders. In this review, we summarize the recent progress made with regards to the function of lncRNAs and associated molecular mechanisms, focusing on neuronal development and neurological disorders.

## Introduction

Over the past several decades, advances in genomic sequencing technology and findings from large-scale consortia have facilitated our understanding of the complexity and flexibility of mammalian genomes. The majority of mammalian genomes are transcribed, whereas only a few transcripts encode proteins, the majority of transcripts are non-coding RNAs (ncRNAs) (Roberts et al., 2014). Based on the length of transcripts, ncRNAs are usually classified into two categories: small non-coding RNAs and long non-coding RNAs (lncRNAs). Small ncRNAs are usually <200 nucleotides, including microRNAs, Piwi-interacting RNAs and small nuclear RNAs (snoRNAs). lncRNAs are >200 nucleotides and frequently transcribed by polymerase II, and share some features, e.g., 5′-capping, 3′-polyadenylation, alternative splicing and sequence conservation with mRNA (Ponting et al., 2009; Nagano and Fraser, 2011).

Although lncRNAs generally lack protein coding capacity, spatiotemporal-specific expression patterns have highlighted the diverse functions and complicated mechanisms of lncRNAs (Cao et al., 2018). Currently, it is widely accepted that lncRNAs play an important function in a variety of biological processes, including regulating gene expression, both at the transcriptional and the post-transcriptional level, shaping the chromatin conformation and imprinting the genomic loci (Lee and Bartolomei, 2013; Chen, 2016; Cao et al., 2018), and multiple diseases such as neurological disorders, cancer, and immunological diseases (Bian and Sun, 2011; Huarte, 2015; Wan et al., 2017). In this review, we summarize the recent progress made regarding the functions of lncRNAs, especially the functions and associated mechanisms related to neurological disorders.

## Characterization of LncRNA

**FIGURE 1:**
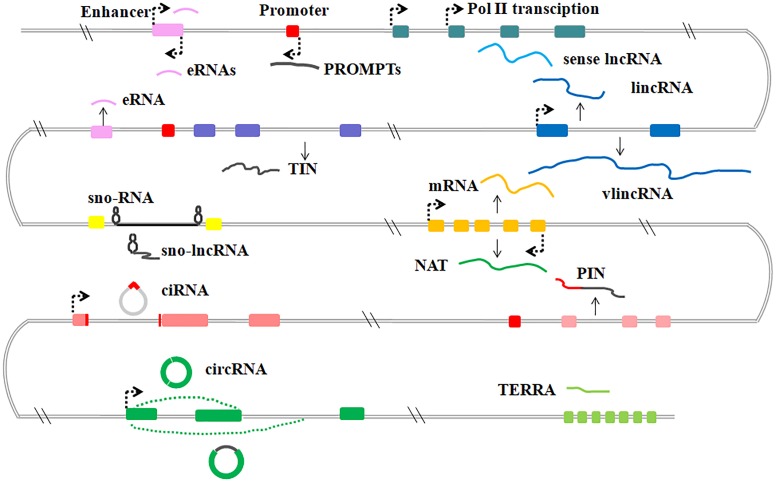
Graphic illustration of the classification of lncRNAs in mammalian. General classification of lncRNAs. eRNAs, enhancer RNAs; PROMPTs, promoter upstream transcripts; lincRNAs, large intergenic ncRNAs; vlincRNA, very long intergenic ncRNAs; TIN, totally intronic long non-coding RNAs; sno-lncRNAs, small nucleolar RNA (snoRNA)-ended lncRNAs; NATs, natural antisense transcripts; PIN, partially intronic lncRNAs; ciRNAs, circular intronic RNAs; circRNAs, circular RNAs; TERRA, telomeric repeat-containing RNA.

LncRNAs are generally transcribed from various genomic contexts and tend to have fewer exons than protein-coding transcripts (Iyer et al., 2015). Although there are still many challenges in annotation and interpretation of lncRNAs, because of the lack of an unambiguous classification framework, the existing lncRNAs can be subdivided into several categories based on their positional relation to protein coding genes, DNA elements or diverse mechanisms of processing (St Laurent et al., 2015; Kopp and Mendell, 2018) (Figure 1).

Sense lncRNAs are transcribed from the sense DNA strand, and have overlapping regions with protein-coding genes, including un-spliced sense partially intronic RNAs (PINs) and spliced transcripts resembling mRNAs (St Laurent et al., 2015). Further, natural antisense transcripts (NATs) of protein-coding genes have also been identified and many NATs share some opposite strand DNA sequences with the sense transcripts (Katayama et al., 2005). Some studies also indicate that NATs have either positive or negative effects on the corresponding sense transcripts or nearby protein-coding transcripts (Faghihi et al., 2010; Modarresi et al., 2012). For example, human brain-derived neurotrophic factor antisense RNA (*BDNF-AS*) was originally identified as natural antisense transcripts of neuronal transcriptional factor BDNF, shares 225 complementary nucleotides with BDNF mRNA and regulates the expression of BDNF both *in vivo* and *in vitro* (Modarresi et al., 2012;Fatemi et al., 2015).

Other studies indicate that intronic regions of coding genes produce a lot of lncRNAs. These intronic lncRNAs form the largest class of lncRNAs and are expressed independently from the pre-mRNA of protein coding genes. Many intronic lncRNAs fail to be debranched after splicing and form a covalent circle without 3′ linear appendages, these circular intronic ncRNAs (ciRNAs) were found to play a regulatory role on their host genes (Zhang et al., 2013). In addition, circRNAs derived from the internal exons of pre-mRNAs through back-splicing, have also been found in various cell lines and tissues (Wu H. et al., 2017). These circular ncRNAs usually present tissue- and developmental stage-specific expression, such as the intensively studied cerebellar degeneration-related protein 1(CDR1as) (Memczak et al., 2013).

A relatively well-characterized subclass of lncRNAs is large/long intergenic or intervening non-coding RNAs (lincRNAs), and transcribed from the intergenic regions. LincRNAs have no overlapping sequences with transcripts of either protein-coding genes or other types of genes (Clark and Blackshaw, 2014). At the molecular level, most annotated lincRNAs have mRNA-like features including 5′-cap structures, 3′-poly(A) tails, exon–exon splice junctions and association with ribosomes (Cabili et al., 2011). Compared with mRNA counterparts, lincRNAs exhibit a more tissue-specific expression, a greater nuclear localization and less evolutionary conservation (Djebali et al., 2012).

Promoter upstream transcripts (PROMPTs) localize in a fairly narrow region between ∼500 and ∼2500 nucleotides upstream of transcription start sites of nearby active protein-coding genes (Preker et al., 2011; Lloret-Llinares et al., 2016). It was reported that the expression levels of certain PROMPTs are altered in stress conditions, such as DNA damage responses and osmotic responses (Lloret-Llinares et al., 2016; Song et al., 2018). Enhancer-related lncRNAs (eRNAs) are bidirectional transcripts of enhancers and have enhancer-like functions. Increased binding of DNA hydroxylase Tet1 and histone methyltransferases Mll3/Mll4 and DNA hypomethylation and H3K27ac modifications at enhancers, may activate eRNAs transcription. Both PROMPTs and eRNAs are targets of the RNA exosome and display similarities during processing (Andersson et al., 2014; Wu H. et al., 2017).

Emerging evidence indicates that telomeric repeat-containing RNA (TERRA) is a heterogeneous lncRNA consisting of a combination of subtelomeric and telomeric sequences. These sequences are mostly transcribed from intrachromosomal telomeric repeats by pol II and polyadenylated at 3′ region (Luke and Lingner, 2009). The length and expression level of human TERRA is influenced by the telomere length. The vast majority of mouse TERRA-binding sites were found in distal intergenic and intronic regions, where TERRA may regulate expression of target genes (Chu et al., 2017; Diman and Decottignies, 2018).

SnoRNA-ended lncRNAs (sno-lncRNAs) are transcripts of one intron flanked by two snoRNA genes that can be further processed to form snoRNA. sno-lncRNAs can be stabilized by snoRNPs formed by snoRNAs and specific protein components. *SLERT* is a representative Box H/ACA snoRNA-ended lncRNA and has been reported to be translocated to the nucleus by snoRNAs to function in pre-rRNA biogenesis (Wu H. et al., 2017).

## Physiological Functions of LncRNA

Loss- and gain-of-function studies revealed that many lncRNAs are involved in various biological processes during development. Many lncRNAs have been found to regulate transcription via chromatin modulation, by working as molecular scaffolds for protein–protein interactions or interacting with chromatin modifying complexes and recruiting chromatin modifying complexes to specific loci, to activate or repress target gene expression. Some lncRNAs could affect transcription by modulating the binding of the general transcription machinery and regulatory factors (Wang and Chang, 2011; Fang and Fullwood, 2016; Wan et al., 2017; Lekka and Hall, 2018). Aside from modulating chromatin states, nuclear lncRNAs are involved in the RNA processing (Tripathi et al., 2010), turnover, silencing, translation and decay of mRNAs (Gong and Maquat, 2011; Carrieri et al., 2012; Geisler and Coller, 2013), or act as miRNA decoys to neutralize miRNA-mediated mRNAs silencing and interact with signaling molecules, to modulate signaling pathways (Faghihi et al., 2010; Liu et al., 2015). In addition, some lncRNAs are determined to be precursors of certain miRNAs at particular stages of development (Dykes and Emanueli, 2017) (Table 1).

**Table 1:** Diverse mechanisms of lncRNAs playing function.

Mechanism	lncRNA	Function	Relationship with target (*Cis/Trans*)	Reference
Chromatin remodeling	ANRIL	Repression at the CDKN2A/B locus	*Cis/trans*	Congrains et al., 2013
	BCAR4	SNIP1 and PNUTS recruitment	*Trans*	Xing Z. et al., 2014
	Braveheart	Activation of *MesP1*	*Trans*	Klattenhoff et al., 2013
	cga eRNA	Formation of looping between enhancer and promoter	*Cis*	Pnueli et al., 2015
	COOLAIR	Repression at the FLC locus	*Cis*	Marquardt et al., 2014
	FENDRR	PRC2 and TrxG/MLL complexes recruitment	*Trans*	Grote et al., 2013
	H19	Genomic imprinting	*Cis/trans*	Cai and Cullen, 2007
	HAUNT	Repression at the HOXA locus	*Cis*	Yin et al., 2015
	HOTAIR	Repression at the HOXD locus	*Trans*	Cai et al., 2014
	HOTTIP	Activation at the HOXA locus	*Cis*	Lian et al., 2016
	HOXD-AS1	Recruitment of WDR5 to target genes	*Trans*	Gu et al., 2017
	Kcnq1ot1	Imprinting at the KCNQ1 cluster	*Cis*	Chiesa et al., 2012
	lncTCF7	Recruitment of SWI/SNF complex to TCF7	*Cis*	Wu B. et al., 2018
	MEG3	Accumulation of p53 protein	*Cis/trans*	Zhou et al., 2007
	Oct4P4	Repression at the Oct4 locus	*Trans*	Scarola et al., 2015
	PAPAS	rRNA synthesis	*Cis*	Zhao Z.L. et al., 2018
	PARTICLE	Repression of methionine adenosyltransferase 2A	*Cis/trans*	O’leary et al., 2017
	TERRA	Felomeric heterochromatin formation	*Cis/trans*	Luke and Lingner, 2009
	TSIX	X inactivation	*Cis*	Sado et al., 2005
	XIST	X inactivation	*Cis*	Cerase et al., 2015
	Six3OS	Recruit histone modification enzymes to Six3 target genes	*Trans*	Rapicavoli et al., 2011
DNA methylation	APTR	Recruitment of PRC2 to CDKN1A/p21	*Trans*	Negishi et al., 2014
	DALI	DNA methylation on promoter regions of target genes	*Trans*	Chalei et al., 2014
	DUM	DNA methylation on Dppa2	*Cis*	Wang et al., 2015a
	Evf2/Dlx6as	Transcriptional repression of Evf2 and Dlx5	*Cis/trans*	Berghoff et al., 2013
	FIRRE	H3K27me3 methylation maintenance	*Trans*	Yang et al., 2015
miRNA binding	CDR1as	miR-7 decoy	*Trans*	Yu et al., 2016
	lincRNA-ROR	miR-145 binding	*Trans*	Wang et al., 2013
	lncRNA-ATB	miR-200s binding	*Trans*	Li et al., 2017
	UCA1	miR-184 sponge	*NA*	Li and Hu, 2015
	TUG1	miR-144/145 binding	*Trans*	Li et al., 2016
Transcriptional regulation	Uph	Repression at the *Hand2* locus	*Cis*	Kopp and Mendell, 2018
	Paupar	Negative regulation Pax6 expression	*Cis/trans*	Vance et al., 2014
	PANDA	Repression of NF-YA-mediated transcription	*NA*	Puvvula et al., 2014
	Airn	Imprinting at the IGF2R cluster	*Cis*	Kopp and Mendell, 2018
	RMST	Transcriptional coregulator of SOX2	*Trans*	Ng et al., 2013a.
Post transcriptional regulation	MALAT1	Ser/Arg splicing factor regulation	*Trans*	Wu Y.T. et al., 2015
	PCA3	PRUNE2 editing and stability	*Cis*	Salameh et al., 2015
	CCAT2	Alternative splicing of Glutaminase (GLS)	*Trans*	Xin Y. et al., 2017
	MIAT	Alternative splicing of DISC1, ERBB4	*Trans*	Sun et al., 2018
	Sirt1 AS	Promotion of *Sirt1* mRNA stability	*Cis*	Wang et al., 2014
	TINCR	Stability of multiple mRNAs	*Trans*	Kretz, 2013
	1/2-sbsRNA	Activation of STAU1-mediated decay	*Trans*	Gong and Maquat, 2011
	BACE1-AS	Positive regulation of BACE1	*Cis*	Modarresi et al., 2011
	aHIF	Nuclear membrane trafficking	*Cis*	Cayre et al., 2003
	AS UCHL1	UCHL1 mRNA translation	*Cis*	Carrieri et al., 2015
	lincRNA-p21	Translational suppression	*Cis*	Dimitrova et al., 2014
	ZEB2 NAT	Activation of ZEB2 translation	*Cis*	Bernardes De Jesus et al., 2018
	NORAD	Inhibition of PUM protein activity	*NA*	Lee et al., 2016
	SNHG4/5/6	Localization of MTA2 protein in the nuclear	*NA*	Chang et al., 2016; Zhao et al., 2016
	LINK-A	Recruitment of BRK to GPNMB	*NA*	Wu D. et al., 2017
	NEAT1	Formation of nuclear paraspeckles	*Trans*	Clemson et al., 2009
	AOC4P	Degradation of vimentin	*Trans*	Wang et al., 2015b
	ASBEL	Localization of ANA/BTG3 mRNA in the nuclear	*Cis*	Zhao J. et al., 2018
	GAS5	Repression of glucocorticoid receptor-mediated transcription	*NA*	Kino et al., 2010


### LncRNAs and Stem Cell Pluripotency and Differentiation

Accumulating evidence suggests that lncRNAs exert critical functions in pluripotency maintenance, reprogramming and lineage differentiation of stem cells (Wang and Chang, 2011; Ghosal et al., 2013). The long intergenic non-protein coding RNA regulator of reprogramming (*lincRNA-ROR*), increases the reprogramming efficiency of human induced pluripotent stem cells (iPSCs) and promotes the maintenance of embryonic stem cells (ESCs) pluripotency (Loewer et al., 2010). Similar to a miRNA sponge, *lincRNA-ROR* forms a regulatory feedback loop with miR-145 and OCT4, SOX2, and NANOG, and regulates ESC pluripotency (Wang et al., 2013). *MIAT* (myocardial infarction associated transcript) is a co-activator of Oct4 and participates in OCT4 and NANOG regulatory networks in mouse ESCs. Loss of *MIAT* reduces the expression of *Oct4, Sox2*, and *Klf4*, and inhibits ESCs proliferation (Sheik Mohamed et al., 2010).

### LncRNAs and Development

Genomic imprinting is an important epigenetic mechanism and is crucial for normal development in mammals. It restricts gene expression on one of the two parental chromosomes in diploid cells and affects both male and female descendants (Barlow and Bartolomei, 2014). *H19*, a maternally expressed 2.3 kb lncRNA, is generated from the highly conserved and imprinted vertebrate gene cluster insulin-like growth factor 2 (*Igf2*)/*H19*. *H19* transcripts are the precursors of miR-675-3p and miR-675-5p (Cai and Cullen, 2007). Before parturition, *H19* slows the growth of the placenta down partially, by down-regulating the RNA binding protein HuR. The decreased HuR cannot block the processing of miR-675, which further decreases the growth regulator *Igf1r* with *Igf2* as its main ligand (Keniry et al., 2012). Another two well-characterized lncRNAs that have been found to regulate genomic imprinting are *Kcnq1ot1* (*KCNQ1* opposite strand transcript 1) and *Airn* (antisense of *IGF2R* non-protein coding RNA), both are paternally expressed and regulate transcriptional silencing through a multilayered silencing pathway (Perry and Ulitsky, 2016).

Besides genomic imprinting, dosage compensation plays a vital role in equalizing the dosage of X-linked genes between males and females in heterogametic species. *Xist* is a 17 ∼ 20 kb lncRNA and transcribed from the X inactivation center. During female development, *Xist* initiates X-chromosome inactivation (XCI), by progressively coating the future inactive X chromosome (Xi) and then utilizing its conserved A-repeat domain to bind PRC2, to form a transcriptionally silent nuclear compartment. The compartment is enriched by H3K27me3 and responsible for the chromosome-wide gene repression in the Xi (Chery and Larschan, 2014). *TSIX*, transcribed from the active chromosome (Xa), represses *Xist* at the early steps of X inactivation (Gendrel and Heard, 2014). Another lncRNA *JPX/ENOX*, which is transcribed from *Jpx/Enox* gene, that resides 10 kb upstream of *Xist*, also involves in XCI through repressing the *TSIX* expression from the Xi and evicting nuclear protein CCCTC-binding factor (CTCF) away from promoter of *XIST* to activate the *XIST* expression from Xi (Sun et al., 2013).

### LncRNAs in Neurodevelopment

A great number of lncRNAs are expressed during neural development and in the brain. Using high-throughput technologies, *in situ* hybridization, microarray analysis and RNA sequencing (RNA-seq), researchers have found that most of the lncRNAs examined (849 out of 1328) are expressed in specific cell types, subcellular compartments and different regions of the brain (Ng et al., 2013b; Shi et al., 2017). LncRNAs display differential expressions across the cortical layers, and region-specific expressions in the subventricular zone, dentate gyrus and olfactory bulb of mice (Belgard et al., 2011; Ramos et al., 2013). Based on a unique custom microarray platform, 8 lncRNAs were identified to be expressed in an age-dependent manner, from 36 surgically resected human neocortical samples, ranging from infancy to adulthood (Lipovich et al., 2014). However, the function of those lncRNAs need to be validated, through loss-of-function assays, RNA-protein association assays or assessments of RNA-chromatin association.

Some lncRNAs are also found to participate in neural cell fate determination, neuronal-glia fate switching and oligodendrocyte elaboration. An antisense transcript of the distal-less homeobox 1 (*Dlx1*), *Dlx1AS*, was discovered to be up-regulated during GABAergic differentiation and down-regulated during oligodendrocyte differentiation (Mercer et al., 2010). A subsequent study found that *Dlx1AS* participated in neurogenesis, implying its function in neuronal differentiation by regulating expression of its homeobox gene neighbors (Ramos et al., 2013). *Evf2*, a cloud-forming *Dlx5/6* ultra-conserved eRNA, influences the formation of GABAergic interneurons in both a mouse and human forebrain. In the developing ventral forebrain, *Evf2* regulates *Dlx5, Dlx6* and glutamate decarboxylase 1 (*Gad1*) expression by recruiting DLX1 and/or DLX2 and methyl CpG binding protein 2 (MeCP2) to specific DNA regulatory elements. GAD1 is an enzyme responsible for catalyzing glutamate to form GABA. *Evf2* mouse mutants reduced GABAergic interneurons in the early postnatal dentate gyrus and hippocampus (Bond et al., 2009; Berghoff et al., 2013; Cajigas et al., 2018).

The vertebrate retina is comprised of three well-organized cell type-specific neuron layers, interconnected by synapses (Ng et al., 2013b). *Six3OS* is the long non-coding opposite strand transcript (lncOST) of the homeodomain factor *Six3*. During mammalian eye development, *Six3* regulates both early eye formation and postnatal retinal cell specification. Knockdown of *Six3OS* leads to a decrease of bipolar cells and an increase of Müller glia, similar to the results in the knockdown of *Six3*. In contrast, overexpression of *Six3OS* decreased syntaxin positive cells. Gene perturbation studies revealed that *Six3OS* participates in retinal cell specification as a molecular scaffold, to regulate *Six3* activity rather than expression (Rapicavoli et al., 2011). *TUG1* (taurine upregulated gene 1), a spliced and polyadenylated lncRNA, is highly conserved in humans and mice. *TUG1* may participate in rod-photoreceptor genesis and inhibits cone-photoreceptor gene expression globally, by altering the chromatin configurations of photoreceptor-specific transcription factors Crx and Nrl (Young et al., 2005).

## LncRNAs in Neurologic Disorders

Emerging evidence has shown that the dysregulation of lncRNAs is related to multiple neurological disorders, such as schizophrenia (Scholz et al., 2010), autism spectrum disorder (ASD) (Wang et al., 2015c), Parkinson’s (Ni et al., 2017), Huntington’s (Sunwoo et al., 2017) and Alzheimer’s diseases (Faghihi et al., 2008).

Schizophrenia (SCZ) is a debilitating mental disorder with a broad spectrum of neurocognitive impairments. Abundant data suggests that both genetic and environmental factors contribute to the pathophysiology of SCZ (Seidman and Mirsky, 2017). Several lncRNAs have been used as biomarkers and therapeutic targets for SCZ (Chen et al., 2016). lncRNA *MIAT*, also known as Gomafu or RNCR2, is down-regulated in SCZ upon neuronal activation (Sun et al., 2018). Previous studies found MIAT either acts as a competitive endogenous RNA (ceRNA) for miR-150-5p, miR-24, miR-22-3p or miR-150, to influence cell proliferation, apoptosis and migration, or participates in various signaling pathways by enhancing Nrf2 (nuclear factor erythroid 2-related factor 2) and Oct4 expression. Subsequent studies revealed that *MIAT* can directly bind to the splicing regulator quaking homolog (QKI) and splicing factor 1 (SF1), to modulate several gene expressions in the neuron. In SCZ patient brains, *DISC1* (disrupted in schizophrenia 1), *ERBB4* (v-erb-a erythroblastic leukemia viral oncogene homolog 4) and their alternatively spliced variants were all down-regulated due to *MIAT* up-regulation. *MIAT* could act as a scaffold to affect alternative splicing of those SCZ-associated genes (Roberts et al., 2014; Liu et al., 2018; Sun et al., 2018).

Autism spectrum disorder (ASD) is a heterogeneous group of neurodevelopmental disorders characterized by impaired reciprocal social interactions, communication, and repetitive stereotyped behaviors (Tang et al., 2017). 222 differentially expressed lncRNAs have been identified from autistic brain tissues. 90% of these lncRNAs are oriented in or around known genes related to neurodevelopmental and psychiatric diseases, such as *UBE3A* (ubiquitin protein ligase E3A), which is associated with Angelman syndrome, that shares common features with ASD. At the same time, it has been found that the number of lncRNAs differentially expressed within a control sample, was much greater than that within an autistic sample (1375 lncRNAs vs. 236 lncRNAs, respectively) (Ziats and Rennert, 2013). A genome-wide association study (GWAS) of ASD identified a 3.9 kb lncRNA designated MSNP1AS, which is encoded by the opposite strand of the moesin pseudogene 1 (*MSNP1*). The sense transcript MSN encodes the moesin protein that regulates neuronal architecture and immune responses. MSNP1AS was found to be significantly upregulated in a postmortem ASD temporal cortex, and overexpression of *MSNP1AS* led to significant decreases in MSN, moesin, neurite number and length in cultured neurons. Thus, *MSNP1AS* contributes to ASD risk, by possibly influencing the sense transcript MSN expression negatively (Wilkinson and Campbell, 2013).

*BACE1-AS* is a conserved non-coding antisense transcript of β-secretase 1 (*BACE1*) and has been shown to be closely associated with Alzheimer’s disease (AD). BACE1 is responsible for the generation of β-amyloid and the amyloid plagues in the brain, which are the primary pathophysiology of AD. *BACE1-AS* is markedly up-regulated in AD brains and promotes the stability of BACE1 through stabilizing BACE1 mRNA, thereby increasing the BACE1 protein and Aβ1–42 levels (Faghihi et al., 2008). Knock down of *BACE1-AS in vivo* resulted in the down-regulation of both *BACE1* and *BACE1-AS*, along with reduced β-amyloid in the brain. In addition, the brain cytoplasmic RNA BC200 (*BCYRN1*), GDNF gene antisense transcript (*GDNF-AS*) and Sox2 overlapping transcript (*Sox2OT*), all participate in progress and development in AD brains (Wan et al., 2017).

Huntington disease (HD) is a hereditary neurodegenerative disease with symptoms including dementia, chorea, and psychiatric disturbances. HD is caused by a CAG trinucleotide abnormal expansion in the first exon of the huntingtin gene and its probability of occurrence is 1/10000. Microarray data found that the expression of four lncRNAs significantly changed in HD brains: *NEAT1* (nuclear paraspeckle assembly transcript 1) and *TUG1* are upregulated, and *DGCR5* (DiGeorge syndrome critical region gene 5) and *MEG3* (maternally expressed 3) are downregulated. The up-regulation of *NEAT1* in HD might contribute to the pathogenic alteration of the transcriptional status, by sequestrating various paraspeckle proteins (Sunwoo et al., 2017), whereas *TUG1* is possibly activated by p53 and then interacts with PRC2, to affect downstream HD-associated genes. DGCR5 and MEG3, are both direct targets of REST. Their down-regulation may result from the aberrant accumulation of REST in the nuclei of striatal neurons in HD (Johnson, 2012; Hwang and Zukin, 2018). Using the whole genome chromatin immunoprecipitation sequencing (ChIP-Seq) method, *HAR1*, a deeply conserved genomic region that is directly bound by REST was confirmed. This region encodes a pair of structured lncRNAs as well, HAR1F and HAR1R. Both HAR1F and HAR1R were downregulated in the striatum of HD patients (Johnson et al., 2010).

Parkinson’s disease (PD) is one of most prevalent neurodegenerative disorders, characterized by progressive impairments of motor abilities caused by the loss of dopamine-producing cells in the brain. Antisense ubiquitin carboxy-terminal hydrolase L1 (*AS-Uchl1*) was discovered to up-regulate the translation of *UchL1* protein at a post-transcriptional level depending on a 5′ overlapping sequence and an embedded inverted SINEB2 sequence (Carrieri et al., 2012). *AS-Uch1* is strongly down-regulated in neurochemical models of PD as a component of the Nurr1-dependent gene network and the subsequent reduced translation of UCHL1 protein, lead to the perturbation of the ubiquitin-proteasome system (Carrieri et al., 2015). *H19* upstream conserved 1 and 2 (*Huc1* and *Huc2*), *lincRNA-p21, MALAT1, SNHG1*, and *TncRNA* were differentially expressed in PD patients (Kraus et al., 2017). As these lncRNAs are associated with synaptogenesis, proliferation and apoptosis, the expression of these lncRNAs precede the course of PD, suggesting they may be biomarkers of PD (Kraus et al., 2017).

## Mechanisms of LncRNAs in Biological Processes

**FIGURE 2:**
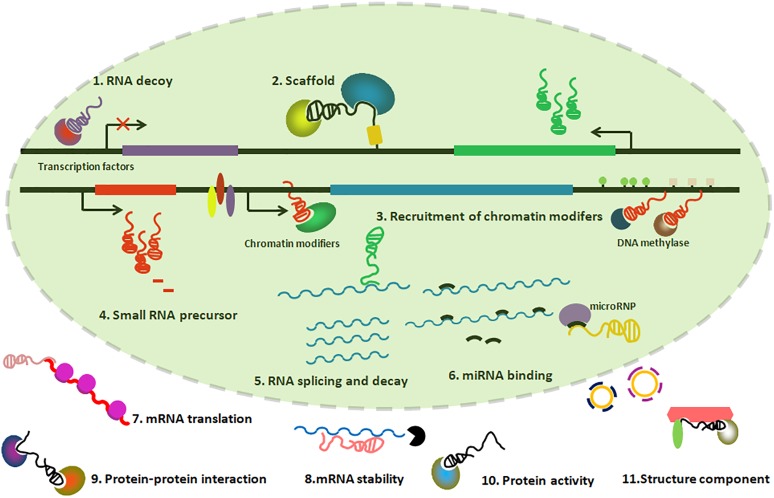
Graphic illustration of Mechanisms of lncRNAs playing functions. (1) lncRNAs can titrate transcription factors away from chromatin; (2) lncRNAs can serve as a scaffold to form ribonucleoprotein complexes; (3) lncRNAs can recruit chromatin-modifying enzymes to target genes; (4) lncRNAs can be precursors of small regulatory RNAs; (5–8) lncRNAs can regulate processes such as RNA splicing, translation and decay, in addition to miRNA binding; (9–11) lncRNAs can participate in protein–protein interactions, regulate protein activity and present as a structural component in the cytoplasm.

lncRNAs could provide functions through differential mechanisms, including serving as molecular scaffolds, molecular signals, guiding chromatin modifiers, and miRNA sponges, etc. (Figure 2).

### Molecular Scaffolds

*Xist*, a 17 ∼ 20 kb lncRNA transcribed from the X inactivation center, initiates X-chromosome inactivation (XCI) by progressively coating the future inactive X chromosome (Xi). *Xist* can bind to chromatin-modifying complexes PRC2 through the conserved A-repeat domain and form a transcriptionally silent nuclear compartment. This compartment is responsible for the chromosome-wide gene repression in the Xi (Chery and Larschan, 2014). Another example of the lncRNA acting as a molecular scaffold is the *HOTAIR*, which are transcripts of the antisense strand of HOXC gene cluster, which can modulate nearby gene expression by interacting with PRC2 and lysine specific demethylase 1 (LSD1) (Tsai et al., 2010).

lncRNA *tsRMST*, an isoform of RMST (rhabdomyosarcoma 2 associated transcript) was highly expressed in human iPSCs and ESCs. Further studies revealed that *tsRMST* down-regulation leads to NANOG and the PRC2 complex component SUZ12 fail to bind to the promoters of several inactive genes. These genes are thereby activated and promote ectoderm and endoderm differentiation (Ng et al., 2012; Yu and Kuo, 2016).

### Molecular Signals

Genomic imprinting restricts gene expression on one of the two parental chromosomes and the parental-specific gene expression in diploid cells, and affects both male and female descendants (Barlow and Bartolomei, 2014). *H19* transcripts are the precursors of miR-675-3p and miR-675-5p (Cai and Cullen, 2007). Before parturition, H19 slows the growth of the placenta down partially, through down-regulating the RNA binding protein HuR. The decreased HuR fails to block the processing of miR-675, which further decreases the growth regulator *Igf1r* (Keniry et al., 2012).

*MALAT1* (metastasis-associated lung adenocarcinoma transcript (1) is initially found as an abundant lncRNA in nuclear speckles to regulate processes of mRNA alternative splicing by modulating the levels of serine/arginine splicing factors (Tripathi et al., 2010). Recent studies confirmed that *MALAT1* is a sensitive prognostic marker for lung cancer metastasis and linked to several other human cancers (Gutschner et al., 2013).

### Guiding Chromatin Modifiers

Genome regulation via DNA methylation and post-translational histone modifications by the activity of chromatin modifiers, is a well-documented function of lncRNA in eukaryotes (Bohmdorfer and Wierzbicki, 2015; Nanda et al., 2016). Altered DNA methylation patterns at CpG islands and mutations in chromatin modifiers, may result in oncogenesis (Haladyna et al., 2015; Nanda et al., 2016).

The first lncRNA identified to interact with both maintenance and *de novo* methylases, *Dum*, is tightly associated with myogenesis and transcriptionally induced by MyoD upon myoblast differentiation. *Dum* can recruit DNA methyltransferase1/3a/3b complex to the *Dppa2* promoter, through intra-chromosomal looping, mediated by RAD21 and NIPBL, resulting in two CpG loci hypermethylation and *Dppa2* silencing (Wang et al., 2015a). Recent studies of *HOTAIR* suggest that under heypoxia, *HOTAIR* expression is up-regulated in several cancer cells induced by the hypoxia-inducible factors (HIFs), recruiting hypoxia-response elements (HRE) to bind on the *HOTAIR* promoter. Along with HIFs, histone acetyltransferase CREB-binding protein (CBP/p300) and histone H3K4 specific methyltransferases, mixed lineage leukemia (MLL) family, are enriched in the HRE region of the *HOTAIR* promoter (Bhan et al., 2017).

Additionally, N-Myc can directly bind to the JMJD1A promoter to upregulate JMJD1A expression in neuroblastoma cells. The upregulated JMJD1A then directly binds to the *MALAT1* promoter to demethylate H3K9, to activate *MALAT1* expression (Tee et al., 2014; Peng et al., 2018). Furthermore, *H19* and mir-675 were found to participate in the adipogenesis through mir-675, targeting the histone deacetylase (HDAC) 4–6 3′ untranslated regions and inducing HDACs 4–6 down-regulation. The reduced HDAC 4–6 then reduced *H19* expression, possibly by reducing the levels of CTCF occupancy in the *H19* imprinting control region. H19 inhibition then facilitates the bone marrow mesenchymal stem cells differentiating into adipocytes (Huang et al., 2016).

### miRNA Sponges

A handful of microRNAs have been reported to influence the mRNA stability of protein-coding genes on post-transcriptional level. Recent studies on lncRNAs discovered several miRNA-lncRNA interactions based on in *silico* and experimental analyses (Paraskevopoulou and Hatzigeorgiou, 2016). One of the well-studied lncRNAs which act as miRNA sponges, is *lincRNA-ROR*, which decoys miR-145 in self-renewing human ESCs. A regulatory feedback loop formed by lincRNA-RoR, miR-145 and the core transcription factors OCT4, SOX2, and NANOG is closely related to the ESCs pluripotency (Wang et al., 2013). *MALAT1* not only interacts with several splicing factors, but also binds to miRNAs including miR-101, miR-9, miR-125b, and miR217 to regulate the interactions between miRNA and mRNAs (Paraskevopoulou and Hatzigeorgiou, 2016).

### Other Mechanisms

Some lncRNAs exert their function by maintaining DNA looping between enhancer and promoter regions, or by recruiting chromatin regulatory proteins to establish high affinity interactions between different regions of the DNA, resulting in closely positioned promoters and enhancers (Yang et al., 2013). For example, *linRNA-p21* regulates the expression of its neighboring gene *p21*, by *cis*-regulatory enhancer-like DNA elements, which embed within the *p21* gene body (Dimitrova et al., 2014). lncRNAs can also interact with DNA directly through nucleic-acid hybridization, and regulate nearby gene expression, such as *Airn* (antisense of IGF2R non-protein coding RNA), which produces transcription interference on paternal allele, by spanning the *Igf2* gene promoter (Latos et al., 2012). Additionally, lncRNAs may influence the three-dimensional organization of the mammalian nucleus. *FIRRE* (firre intergenic repeating RNA element) is transcribed from the X chromosome, and interacts with hnRNPU in the process of nuclear organization (Yang et al., 2015). *Trans*-acting lncRNAs also modulate the protein activity and RNA stability by directly binding in the nucleus or cytoplasm. lncRNA NORAD functions as a negative regulator of the RNA binding protein PUMILIO1 and 2 (PUM1 and 2) in the cytoplasm. The knockdown of NORAD leads to the degradation of PUM1/2 targeted mRNAs (Lee et al., 2016).

## Perspectives

Previous studies have revealed that lncRNAs play an important role in neuronal development and function through differential mechanisms. The dysregulation of lncRNAs could result in neurological diseases. Advances in sequencing technologies and their applications will contribute substantially to uncovering and investigating novel lncRNAs and their functions.

One challenge in the lncRNA field is whether lncRNAs can be used as diagnostic biomarkers or therapeutic targets for diseases. Considering that the down-stream targets of lncRNAs could be broad, it is hard to use it as a specific “key” to one “lock.” Although the validation of their functions could be performed *in vitro* and *in vivo*, it is difficult to claim a specific target. Another challenge is to better understand the mechanisms of lncRNAs functions. It is highly necessary to develop proper genetic tools and to establish animal models to dissect the regulatory networks of lncRNAs, and their interaction with other epigenetic modifications.

## Author Contributions

LL, YZ, XZ, and XL wrote the manuscript. All authors commented on the manuscript.

## Conflict of Interest Statement

The authors declare that the research was conducted in the absence of any commercial or financial relationships that could be construed as a potential conflict of interest.
